# Supplementary material for: Anti-CRISPR Anopheles mosquitoes inhibit gene drive spread under challenging behavioural conditions in large cages
Source: Nat Commun. 2024 Feb 1;15:952. doi: 10.1038/s41467-024-44907-x (PMC10830555; doi:10.1038/s41467-024-44907-x)
Supplement: Supplementary file 1 — Supplementary Information [file 41467_2024_44907_MOESM1_ESM.pdf]

SUPPLEMENTARY INFORMATION

Title

Anti-CRISPR *Anopheles* mosquitoes inhibit gene drive spread under challenging behavioural conditions in large cages

Authors

Rocco D'Amato[1], Chrysanthi Taxiarchi[2], Marco Galardini[3,4,5], Alessandro Trusso[1], Roxana L. Minuz[1], Silvia Grilli[2], Alastair GT Somerville[2], Dammy Shittu[2], Ahmad S. Khalil[3,6,7], Roberto Galizi[8], Andrea Crisanti[2,9], Alekos Simoni[1*§], Ruth Müller[1,10*§]

Affiliations

[1] Genetics and Ecology Research Centre, Polo of Genomics, Genetics and Biology (Polo GGB), Terni, Italy

[2] Department of Life Sciences, Imperial College London, London, United Kingdom

[3] Biological Design Center, Boston University, Boston, MA, USA

[4] Institute for Molecular Bacteriology, TWINCORE Centre for Experimental and Clinical Infection Research, a joint venture between the Hannover Medical School (MHH) and the Helmholtz Centre for Infection Research (HZI), Hannover, Germany

[5] Cluster of Excellence RESIST (EXC 2155), Hannover Medical School (MHH), Hannover, Germany

[6] Department of Biomedical Engineering, Boston University, Boston, MA, USA

[7] Wyss Institute for Biologically Inspired Engineering, Harvard University, Boston, MA, USA

[8] Centre for Applied Entomology and Parasitology, School of Life Sciences, Keele University, Keele, UK

[9] Department of Molecular Medicine, University of Padova, Padua, Italy

[10] Unit of Entomology, Department of Biomedical Sciences, Institute of Tropical Medicine, Antwerp, Belgium

SUPPLEMENTARY TABLES

Supplementary Table 1. Characterisation of selected transgenic founders carrying (Vasa:A4)2 transgene insertion in trans-heterozygosity with (QFS)1. The final column shows the inheritance rate of the (Vasa:A4)2 transgene scored in the progeny. The total number of larvae screened is given in parentheses.

Founder	(Vasa:A4)2 transgene genomic insertion		(Vasa:A4)2 inheritance rate
	genomic location	Sequencing method	%
♀ 1	AGAP012655 (UNK) / AGAP009548 (3R) exon 2	Inverse PCR	64.2 (95)
♀ 2	AGAP012655 (UNK) / AGAP009548 (3R) exon 2	Inverse PCR	44.3 (106)
♂ 1	AGAP012655 (UNK) / AGAP009548 (3R) exon 2	Inverse PCR	55.1 (118)
♂ 2	AGAP004649 (2R) intron 1	Nanopore	56.3 (144)

Supplementary Table 2. Time of pupation of mosquitoes carrying one or two copies of the Ag(Vasa:A4)2 construct. Scoring of the male and the female pupae collected every day per genotype. Each percentage value represents the average from three biological replicates, each of them consisting of three replicates. Statistical analysis has been performed comparing separately the total number of males and females pupae for each sex genotype with the corresponding wild-type individual, with no differences highlighted (Ag(Vasa:A4)$2^{+/-}$ males p=0.88, Ag(Vasa:A4)$2^{+/-}$ females p=0.89, Ag(Vasa:A4)$2^{+/+}$ males p=0.22, Ag(Vasa:A4)$2^{+/+}$ females p=0.88; one-side *Mann-Whitney test*)

**Days**	**♂ G3**	**♀ G3**	**♂ Ag(Vasa:A4)$2^{+/-}$**	**♀ Ag(Vasa:A4)$2^{+/-}$**	**♂ Ag(Vasa:A4)$2^{+/+}$**	**♀ Ag(Vasa:A4)$2^{+/+}$**
**1**	20.22 (± 11.07)	18.74 (± 12.02)	22.38 (± 11.92)	19.83 (± 10.30)	18.75 (± 9.76)	18.92 (± 9.10)
**2**	20.67 (± 11.07)	22.64 (± 12.55)	17.36 (± 13.00)	18.03 (± 14.51)	18.11 (± 12.20)	18.23 (± 12.26)
**3**	12.77 (± 9.03)	12.15 (± 8.55)	11.91 (± 11.00)	13.35 (± 10.04)	11.65 (± 7.71)	12.56 (± 9.67)
**4**	5.65 (± 3.77)	8.73 (± 3.77)	2.69 (± 1.93)	4.25 (± 3.24)	8.47 (± 4.51)	7.77 (± 4.55)

Supplementary Table 3. **Larval and pupal survival.** Larvae and pupae from Ag(Vasa:A4)$2^{+/-}$, Ag(Vasa:A4)$2^{+/+}$ and G3 strains were recorded and the average of the three biological replicates (three technical replicates each) was calculated (see Data Source file). In addition, aquatic survival probability, calculated by multiplying larval to pupal survival rate, was evaluated for the two anti-drive genotypes and no statistical differences were highlighted when compared to the wild-type sample (Ag(Vasa:A4)$2^{+/-}$ p= 0.38, Ag(Vasa:A4)$2^{+/+}$ p> 0.99; one-side *Kruskal-Wallis's* test).

Genotype	Average larval survival rate	Average pupal Survival rate	Aquatic survival Probability
**G3**	91 (± 0.08)	93 (± 0.03)	85 (± 0.09)
**Ag(Vasa:A4)2+/-**	93 (± 0.18)	93 (± 0.04)	74 (± 0.16)
**Ag(Vasa:A4)2+/+**	84 (± 0.19)	94 (± 0.04)	76 (± 0.18)

Supplementary Table 4. Mating probability. G3 females were crossed *en masse* to Ag(Vasa:A4)$2^{+/-}$, Ag(Vasa:A4)$2^{+/+}$ or G3 males to measure the probability of mating in medium-sized cages, in three biological replicates. Statistical difference was observed for the Ag(Vasa:A4)$2^{+/+}$ (p=0.0407; one-side *Kruskal-Wallis's test*).

**Crossing genotype**	**Mating probability**	
	**% (± s.d.)**	**P**
♂ G3 x ♀ G3	59 (± 4.36)	**-**
♂ Ag(Vasa:A4)$2^{+/-}$ x ♀ G3	52 (± 6.56)	0.821
♂ Ag(Vasa:A4)$2^{+/+}$ x ♀ G3	41 (± 7)	0.041

Supplementary Table 5. Pooled amplicon sequencing of 'gene drive only' population from large-sized cage. The sequences listed, containing single SNPs close to the Cas9/gRNA cleavage site (bolded and depicted in red), come out from amplicon sequencing of sample from 'gene drive only' populations. The naming of each mutation indicates the base pair changed and its location relative to the reference sequence. The % frequency of the indels was detected and analysed at different time points (days post release) from gene drive population, resulting below the frequency of 0.23%.

Gene Drive Population

Indel	Sequence 5' - 3'	Days post release (Indel Frequency %)			
		4	21	49	84
Reference	TCCATTCATTTATGTTTAACACAGGTCAAG|CGGTGGTCAACGAATACTCACGATTGCATA				
30-T-31	TCCATTCATTTATGTTTAACACAGGTCAAG|**T**GGTGGTCAACGAATACTCACGATTGCATA	0.197	0.133	0.118	0.219
22-G-23	TCCATTCATTTATGTTTAACAC**G**GGTCAAG/TGGTGGTCAACGAATACTCACGATTGCATA	0.140	0.082	0.092	0.105
40-T-41	TCCATTCATTTATGTTTAACACAGGTCAAG|CGGTGGTCAA**T**GAATACTCACGATTGCATA	-	0.088	0.064	0.075
41-A-42	TCCATTCATTTATGTTTAACACAGGTCAAG|CGGTGGTCAAC**A**AATACTCACGATTGCATA	-	0.059	0.056	0.144
50-T-51	TCCATTCATTTATGTTTAACACAGGTCAAG|CGGTGGTCAACGAATACTCA**T**GATTGCATA	-	0.097	0.050	0.129
55-A-56	TCCATTCATTTATGTTTAACACAGGTCAAG|CGGTGGTCAACGAATACTCACGATT**A**CATA	0.141	0.153	0.121	0.224
56-T-57	TCCATTCATTTATGTTTAACACAGGTCAAG|CGGTGGTCAACGAATACTCACGATTG**T**ATA	0.137	0.077	0.057	0.237

Supplementary Table 6. Pooled amplicon sequencing of 'gene drive + Anti-drive' population from large-sized cage. The sequences listed, containing single SNPs close to the Cas9/gRNA cleavage site (bolded and depicted in red), come out from amplicon sequencing of sample from 'gene drive + anti-drive' population. The naming of each mutation indicates the base pair changed and its location relative to the reference sequence. The % frequency of indels was detected and analysed at different time points (days post release) from gene drive + anti-drive populations, resulting below the frequency of 0.29%.

Gene drive + Anti-drive population

<table>
<tr><th rowspan="2">Indel</th><th rowspan="2">Sequence 5' - 3'</th><th colspan="8">Days post release (Indel Frequency %)</th></tr>
<tr><th>4</th><th>21</th><th>49</th><th>84</th><th>105</th><th>203</th><th>231</th><th>273</th></tr>
<tr><td>Reference</td><td>TCCATTCATTTATGTTTAACACAGGTCAAG|CGGTGGTCAACGAATACTCACGATTGCATA</td><td></td><td></td><td></td><td></td><td></td><td></td><td></td><td></td></tr>
<tr><td>30-T-31</td><td>TCCATTCATTTATGTTTAACACAGGTCAAG|TGGTGGTCAACGAATACTCACGATTGCATA</td><td>0.102</td><td>0.155</td><td>0.075</td><td>0.175</td><td>0.239</td><td>0.168</td><td>0.094</td><td>0.194</td></tr>
<tr><td>22-G-23</td><td>TCCATTCATTTATGTTTAACACGGGTCAAG/CGGTGGTCAACGAATACTCACGATTGCATA</td><td>0.127</td><td>0.140</td><td>0.065</td><td>0.135</td><td>0.145</td><td>0.139</td><td>0.208</td><td>0.133</td></tr>
<tr><td>40-T-41</td><td>TCCATTCATTTATGTTTAACACAGGTCAAG|CGGTGGTCAATGAATACTCACGATTGCATA</td><td>0.134</td><td>0.069</td><td>0.067</td><td>0.154</td><td>0.120</td><td>0.235</td><td>0.111</td><td>0.069</td></tr>
<tr><td>41-A-42</td><td>TCCATTCATTTATGTTTAACACAGGTCAAG|CGGTGGTCAACAAATACTCACGATTGCATA</td><td>0.137</td><td>0.073</td><td>0.046</td><td>0.063</td><td>0.131</td><td>0.145</td><td>0.045</td><td>0.049</td></tr>
<tr><td>50-T-51</td><td>TCCATTCATTTATGTTTAACACAGGTCAAG|CGGTGGTCAACGAATACTCATGATTGCATA</td><td>0.112</td><td>0.078</td><td>0.090</td><td>0.111</td><td>0.105</td><td>0.060</td><td>0.111</td><td>0.058</td></tr>
<tr><td>55-A-56</td><td>TCCATTCATTTATGTTTAACACAGGTCAAG|CGGTGGTCAACGAATACTCACGATTACATA</td><td>0.275</td><td>0.270</td><td>0.144</td><td>0.194</td><td>0.260</td><td>0.256</td><td>0.290</td><td>0.232</td></tr>
<tr><td>56-T-57</td><td>TCCATTCATTTATGTTTAACACAGGTCAAG|CGGTGGTCAACGAATACTCACGATTGTATA</td><td>0.118</td><td>0.089</td><td>0.057</td><td>0.237</td><td>0.133</td><td>0.105</td><td>0.116</td><td>0.252</td></tr>
</table>

Supplementary Table 7. List of primers used in this study. Cloning overhangs are underlined with a single line. * Primers used for amplicon sequencing (Illumina adaptors in bold and italicised).

Primer Name	Sequence 5'-3'
RG964	TGTGGCCTGCAGGATGTAGAACGCGAGCAAATTCTTTTCC
RG965	CCCATGGGACCCCGAAAATGTGGCCATTAACAGCAGT
RG966	GGCCACATTTTCGGGGTCCCATGGGTGAGGTG
RG967	ACATCCTGCAGGCCACAATGGTTAATTCGAGCTCG
RG968	CTTCCTACTGCAGGCCCCAACTGGGGTAACCTTT
RG969	CCCCAACTGGGGTAACCTTTGAGTTCTCTCAGTTGGGGGCGAAAATGTGGCCATTAACAGC
RG970	GTTACCCCAGTTGGGGGATCTCGGATCTGACAATGTTCAGT
RG971	CCCCAGTTGGGGCCTGCAGTAGGAAGACGAATAGGTG
NLS-probe	AAAAAGAGGAAGGTGAGCGGCGG
GFP-probe	GACGAGCTGTACAAGTAAAGCGG
* 4050-Illumina-F	***TCGTCGGCAGCGTCAGATGTGTATAAGAGACAG***ACTTATCGGCATCAGTTGCG
* 4050-Illumina-R	***GTCTCGTGGGCTCGGAGATGTGTATAAGAGACAG***GTGAATTCCGTCAGCCAGCA

Supplementary Table 8. Parameters used for medium-sized cage modelling. "W" indicates the wild-type allele at the drive (left) or anti-drive locus (right). "A" indicates the anti-drive allele. "D" indicates the drive allele. "R" indicates alleles causing non-functional resistance to the drive. Aquatic survival probability is assumed to be equal for all the genotypes with no statistical differences. Adult survival values are based on the males and females wild-type data and is the same for all the genotypes. [1] Average values obtained from phenotypic analysis performed in this work. [2] This value is obtained assuming drive and anti-drive loci to be equal to WW AA. [3] This value is obtained assuming drive and anti-drive loci to be equal to WW WW. [4] This value is obtained assuming drive and anti-drive loci to be equal to WW WA. [5] This value is obtained assuming drive and anti-drive loci to be equal to WD WW. [6] This value is obtained assuming gene drive and anti-drive loci to be equal to WD WA. [7] This value is obtained from Kyrou *et al*. *2018*.

Drive locus	Anti-d. locus	Relative mating probability to WW	Laying fitness	Eggs per female relative to wt	Hatching probability	Aquatic survival probability	Adult survival (mean) days	Drive inheritance	Anti-drive inheritance
**Males**									
WW	WW	1 [1]	0.95 [1]	1 [1]	1 [1]	0.84	14	0	0
WW	WA	0.94 [1]	0.97 [1]	1.35 [1]	1.1 [1]	0.84	14	0	0.48
WW	AA	0.69 [1]	0.79 [1]	1.25 [1]	1.05 [1]	0.84	14	0	1
WD	WW	0.86 [1]	0.94 [1]	1.1 [1]	1.06 [1]	0.84	14	0.96	0
WD	WA	1.06 [1]	0.89 [1]	1.07 [1]	1.1 [1]	0.84	14	0.52	0.51
WD	AA	0.69 [2]	0.79 [2]	1.07 [6]	1.1 [6]	0.84	14	0.52	1
WR	WW	1 [3]	0.94 [5]	1 [3]	1 [3]	0.84	14	0	0
WR	WA	0.94 [4]	0.89 [6]	1.35 [4]	1.1 [4]	0.84	14	0	0.48 [4]
WR	AA	0.69 [2]	0.79 [2]	1.25 [2]	1.05 [2]	0.84	14	0	1
DD	WW	0.86 [5]	0.94 [5]	1.1 [5]	1.06 [5]	0.84	14	1	0
DD	WA	1.06 [6]	0.89 [6]	1.07 [6]	1.1 [6]	0.84	14	1	0.48 [4]
DD	AA	0.69 [2]	0.79 [2]	1.07 [6]	1.1 [6]	0.84	14	1	1
DR	WW	0.86 [5]	0.94 [5]	1.1 [5]	1.06 [5]	0.84	14	0.5	0
DR	WA	1.06 [1]	0.89 [6]	1.07 [6]	1.1 [6]	0.84	14	0.5	0.48 [4]
DR	AA	0.69 [2]	0.79 [2]	1.07 [6]	1.1 [6]	0.84	14	0.5	1
RR	WW	1 [3]	0.94 [3]	1 [3]	1 [3]	0.84	14	0	0
RR	WA	0.94 [4]	0.89 [4]	1.35 [4]	1.1 [4]	0.84	14	0	0.48 [4]
RR	AA	0.69 [2]	0.79 [2]	1.25 [2]	1.05 [2]	0.84	14	0	1
**Females**									
WW	WW	1 [1]	0.95 [1]	1 [1]	1 [1]	0.84	21	0	0
WW	WA	1.19 [1]	1 [1]	1.15 [1]	1.05 [1]	0.84	21	0	0.52 [1]
WW	AA	1 [3]	0.90 [4]	1.08 [1]	1.15 [1]	0.84	21	0	1 [1]
WD	WW	0.13 [1]	0.50 [1]	0.65 [7]	0.62 [7]	0.84	21	0.99 [7]	0
WD	WA	0.64 [1]	0.58 [1]	0.96 [1]	0.81 [1]	0.84	21	0.55 [1]	0.52 [1]
WD	AA	0.64 [6]	0.58 [6]	0.96 [6]	0.81 [6]	0.84	21	0.55 [6]	1
WR	WW	1 [1]	0.50 [5]	1 [3]	1 [3]	0.84	21	0	0
WR	WA	1.19 [5]	0.58 [6]	1.15 [4]	1.05 [4]	0.84	21	0	0.52 [4]
WR	AA	1 [3]	0.58 [2]	1.08 [2]	1.15 [2]	0.84	21	0	1
DD	WW	0	0	0	0	0.84	21	1	0
DD	WA	0	0	0	0	0.84	21	1	0.52 [4]
DD	AA	0	0	0	0	0.84	21	1	1
DR	WW	0	0	0	0	0.84	21	0.5	0
DR	WA	0	0	0	0	0.84	21	0.5	0.52 [4]
DR	AA	0	0	0	0	0.84	21	0.5	1
RR	WW	0	0	0	0	0.84	21	0	0
RR	WA	0	0	0	0	0.84	21	0	0.52 [4]
RR	AA	0	0	0	0	0.84	21	0	1

Supplementary Table 9. Parameters used for large-sized cage modelling. "W" indicates the wild-type allele at the drive (left) or anti-drive locus (right). "A" indicates the anti-drive allele. "D" indicates the drive allele. "R" indicates alleles causing non-functional resistance to the drive. Aquatic survival probability is assumed to be equal for all the genotypes with no statistical differences. Adult survival value is based on the wild-type data and is the same for all the male and female genotypes. (1) Average values obtained from phenotypic analysis performed in this work. (2) This value is obtained assuming drive and anti-drive loci to be equal to WW AA. (3) This value is obtained assuming drive and anti-drive loci to be equal to WW WW. (4) This value is obtained assuming drive and anti-drive loci to be equal to WW WA. (5) This value is obtained assuming drive and anti-drive loci to be equal to WD WW. (6) This value is obtained assuming gene drive and anti-drive loci to be equal to WD WA. (7) This value is obtained from *Kyrou et al. 2018*

Drive locus	Anti-d. locus	Relative mating probability to WW	Fitness	Eggs per female relative to wt	Hatching probability	Aquatic survival probability	Adult survival (mean) days	Drive inheritance	Anti-drive inheritance
**Males**									
WW	WW	1 (1)	0.0478	1 (1)	1 (1)	0.84	6.213	0	0
WW	WA	0.94 (1)	0.0478	1.35 (1)	1.1 (1)	0.84	6.213	0	0.48 (1)
WW	AA	0.69 (1)	0.0478	1.25 (1)	1.05 (1)	0.84	6.213	0	1 (1)
WD	WW	0.86 (1)	0.0478	1.1 (1)	1.06 (1)	0.84	6.213	0.96 (7)	0
WD	WA	1.06 (1)	0.0478	1.07 (1)	1.1 (1)	0.84	6.213	0.52 (1)	0.51 (4)
WD	AA	0.69 (2)	0.0478	1.07 (5)	1.1 (6)	0.84	6.213	0.52 (6)	1
WR	WW	1 (3)	0.0478	1 (3)	1 (3)	0.84	6.213	0	0
WR	WA	0.94 (4)	0.0478	1.35 (4)	1.1 (4)	0.84	6.213	0	0.48 (4)
WR	AA	0.69 (2)	0.0478	1.25 (4)	1.05 (2)	0.84	6.213	0	1
DD	WW	0.86 (5)	0.0478	1.1 (5)	1.06 (5)	0.84	6.213	1	0
DD	WA	1.06 (6)	0.0478	1.07 (5)	1.1 (6)	0.84	6.213	1	0.48 (4)
DD	AA	0.69 (2)	0.0478	1.07 (5)	1.1 (6)	0.84	6.213	1	1
DR	WW	0.86 (5)	0.0478	1.1 (5)	1.06 (5)	0.84	6.213	0.5	0
DR	WA	1.06 (1)	0.0478	1.07 (6)	1.1 (6)	0.84	6.213	0.5	0.48 (4)
DR	AA	0.69 (2)	0.0478	1.07 (6)	1.1 (6)	0.84	6.213	0.5	1
RR	WW	1 (3)	0.0478	1 (3)	1 (3)	0.84	6.213	0	0
RR	WA	0.94 (4)	0.0478	1.35 (4)	1.1 (4)	0.84	6.213	0	0.48 (4)
RR	AA	0.69 (2)	0.0478	1.25 (2)	1.05 (2)	0.84	6.213	0	1
**Females**									
WW	WW	1 (1)	0.0478	1 (1)	1 (1)	0.84	6.213	0	0
WW	WA	1.19 (1)	0.0478	1.15 (1)	1.05 (1)	0.84	6.213	0	0.52 (1)
WW	AA	1 (1)	0.0478	1.08 (1)	1.15 (1)	0.84	6.213	0	1 (1)
WD	WW	0.13 (1)	0.0478	0.65 (7)	0.62 (7)	0.84	6.213	0.99 (7)	0
WD	WA	0.64 (1)	0.0478	0.96 (1)	0.81 (1)	0.84	6.213	0.55 (1)	0.52 (1)
WD	AA	0.64 (6)	0.0478	0.96 (6)	0.81 (6)	0.84	6.213	0.55 (6)	1
WR	WW	1 (1)	0.0478	1 (3)	1 (3)	0.84	6.213	0	0
WR	WA	1.19 (4)	0.0478	1.15 (4)	1.05 (4)	0.84	6.213	0	0.52 (4)
WR	AA	1 (2)	0.0478	1.08 (2)	1.15 (2)	0.84	6.213	0	1
DD	WW	0	0.0478	0	0	0.84	6.213	1	0
DD	WA	0	0.0478	0	0	0.84	6.213	1	0.52 (4)
DD	AA	0	0.0478	0	0	0.84	6.213	1	1
DR	WW	0	0.0478	0	0	0.84	6.213	0.5	0
DR	WA	0	0.0478	0	0	0.84	6.213	0.5	0.52 (4)
DR	AA	0	0.0478	0	0	0.84	6.213	0.5	1
RR	WW	0	0.0478	0	0	0.84	6.213	0	0
RR	WA	0	0.0478	0	0	0.84	6.213	0	0.52 (4)
RR	AA	0	0.0478	0	0	0.84	6.213	0	1

Supplementary Table 10. Fitting of estimated and experimental fitness parameters to the regression model. R^2 values for goodness of fit comparing the experimental data of wild-type, gene drive and anti-drive frequency to simulations assuming different fitness parameter estimates (as indicated) for the large cage population. The average and the standard deviation for the fit of 50 simulations is indicated (in ascending order for the mean). 'Mating: assuming different probability of mating for the anti-drive individual, except when the drive allele is present in the same individual. 'Experimental': parameters as calculated experimentally (as indicated in Supplementary Table 9).

**Fitness estimates**	**Mean R^2**	**R^2 standard deviation**
Mating 0.01	0.707	0.183
Mating 1.00	0.721	0.033
Mating 0.90	0.728	0.023
Mating 0.70	0.743	0.029
Mating 0.50	0.752	0.041
Experimental	0.753	0.034
Mating 0.05	0.782	0.112
Mating 0.30	0.785	0.052
Mating 0.20	0.815	0.043

SUPPLEMENTARY FIGURES

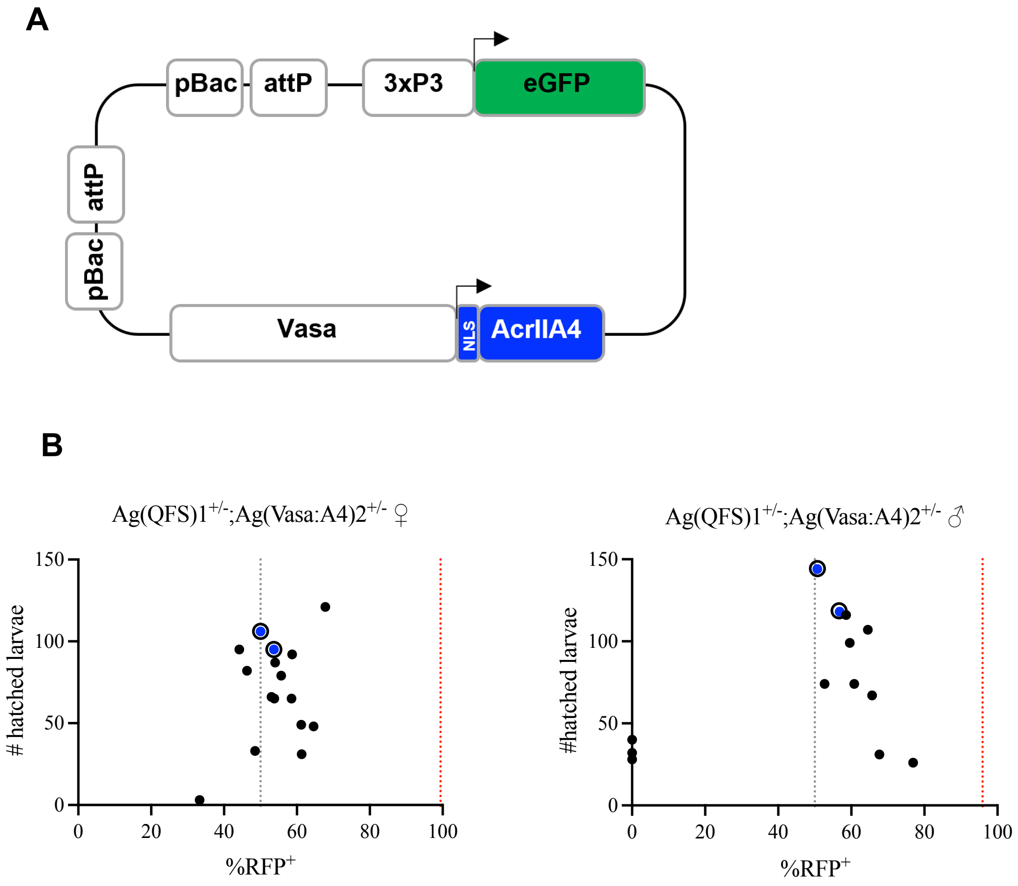

Supplementary Figure 1. Generation and selection of the Ag(Vasa:A4)2 transgenic line. (A) Schematic representation of the construct used to generate the Ag(Vasa:A4)2 line; the construct carries the *Listeria monocytogenes* anti-CRISPR protein (AcrIIA4) expressed under the *vasa* male and female germline-specific promoter with the N-terminus addition of a nuclear localisation signal (NLS) and the eGFP fluorescent protein under the *3xP3* promoter (3xP3:eGFP) used for the screening of anti-drive positive larvae. The construct contains *PiggyBac* repeats on either side for semi-random integration in the genome. **(B)** Fertility and inhibitory activity against Ag(QFS)1 in female (left) or male (right) trans-heterozygote parents, presented as number of hatched larvae per parent against % of larvae that carry the gene drive construct (RFP+) in the progeny of each parent. Blue circled dots represent the progenies selected for further phenotypic analysis. Red dotted lines represent the expected mean gene drive inheritance rate in the absence of anti-CRISPR protein of 99% from females and 96 from males based on data from Kyrou et al 2018. Grey dotted lines represent Mendelian inheritance (50%). Larvae were screened from 16 single cup deposition coming from transgenic females and 13 from transgenic males.

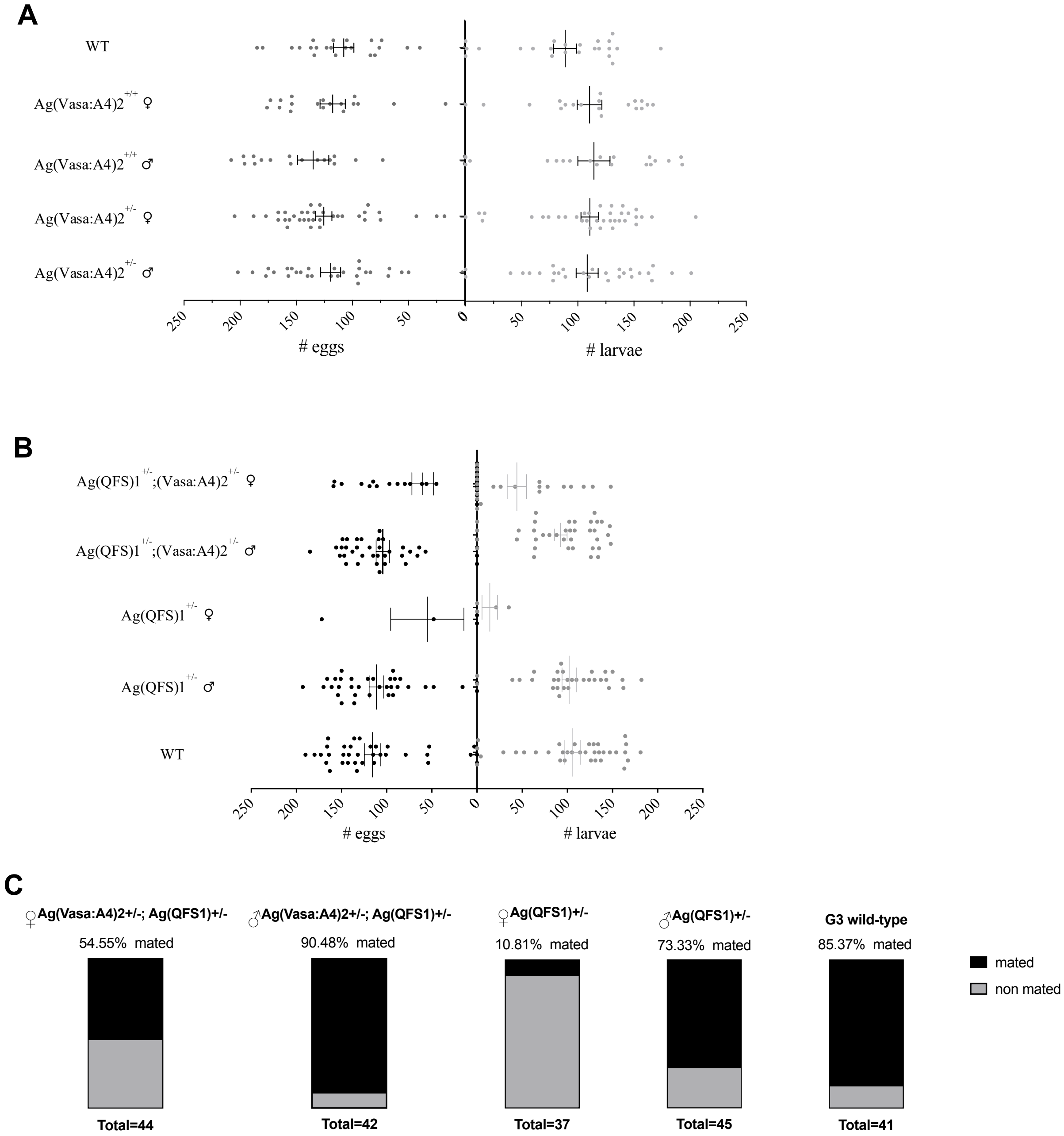

Supplementary Figure 2. Fertility assays and mating rate of gene drive and anti-drive transgenic lines. Scatter plots of the total number of eggs (dark grey dots) and larvae (light grey dots) counted from individual oviposition assays from wild type mosquitoes crossed to transgenic females or males carrying: (**A**) one or two copies of the anti-drive construct (homozygosity or heterozygosity); (**B**) one copy of the gene drive and/or anti-drive constructs (trans heterozygous individuals). Error bars indicate mean values and standard error of the mean of number of eggs or larvae from all biological replicates assessed for each cross. No statistical differences on the number of eggs, when compared the different genotypes with the wild type individuals (p>0.99 for Ag(Vasa:A4)$2^{+/-}$ males, p=0.38 for Ag(Vasa:A4)$2^{+/-}$ females, p=0.094 for Ag(Vasa:A4)$2^{+/+}$ males, p>0.99 for Ag(Vasa:A4)$2^{+/+}$ females; one-side *Kruskal-Wallis's test*), as well as for the number of larvae (p=0.45 for Ag(Vasa:A4)$2^{+/-}$ males, p=0.31 for Ag(Vasa:A4)$2^{+/-}$ females, p=0.30 for Ag(Vasa:A4)$2^{+/+}$ males, p=0.44 for Ag(Vasa:A4)$2^{+/+}$ females; one-side *Kruskal-Wallis's test*) (Data source file contain

the number of single cup deposition for each cross tested in the experiment) (**C**) Mating rate of males and females expressing both gene drive and anti-drive transgene is compared to individuals with only gene drive transgene. Wild type is used as positive control. The 'total' value represents the sum of mated and not mated females.

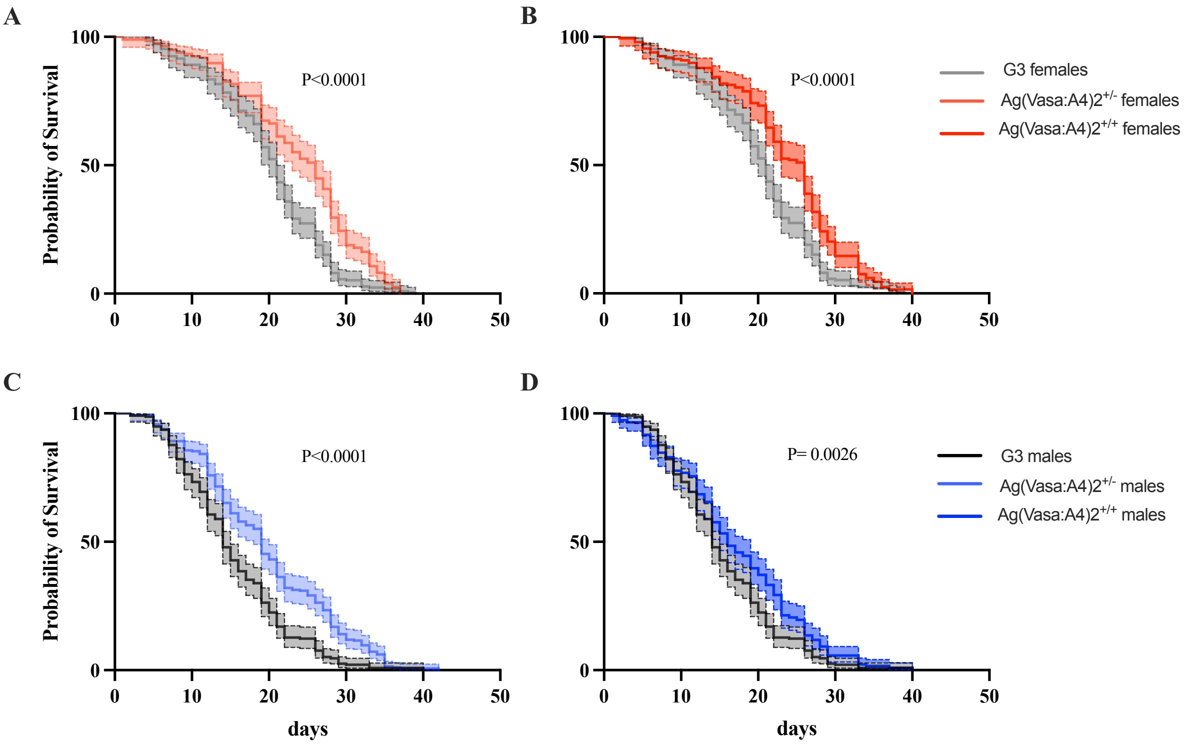

Supplementary Figure 3. Adult survival curves for wild-type (G3) and anti-drive Ag(Vasa:A4)2$^{+/-}$ and Ag(Vasa:A4)2$^{+/+}$ mosquitoes in medium-sized cages. G3, Ag(Vasa:A4)2$^{+/-}$ and Ag(Vasa:A4)2$^{+/-}$ adults were monitored for daily survival in medium-sized cages. Three independent biological replicates were performed and grouped in a unique graph where each genotype is compared with wild type within the same sex. Error bands (coloured shadings) show 95% Confidence Intervals of the mean. Statistical difference between groups was calculated using the *Kaplan-Meier test*.

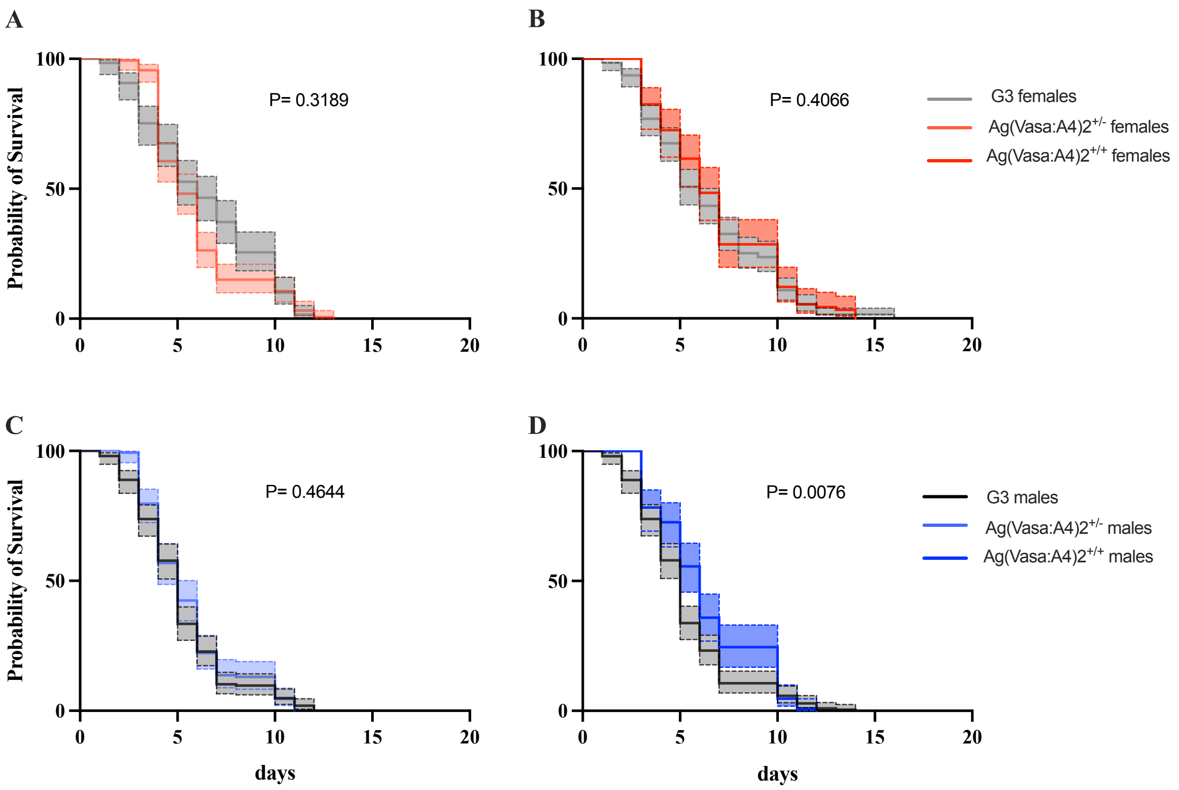

Supplementary Figure 4. Adult survival curves for wild-type (G3) and anti-drive Ag(Vasa:A4)2$^{+/-}$ and Ag(Vasa:A4)2$^{+/+}$ mosquitoes in large-sized cages. G3, Ag(Vasa:A4)2$^{+/-}$ and Ag(Vasa:A4)2$^{+/-}$ adults were monitored for daily survival in large-sized cages. Three independent biological replicates were performed and grouped in a unique graph where each genotype is compared with wild type within the same sex. Error bands (coloured shadings) show 95% Confidence Intervals of the mean. Statistical difference between groups was calculated using the *Kaplan-Meier test*.

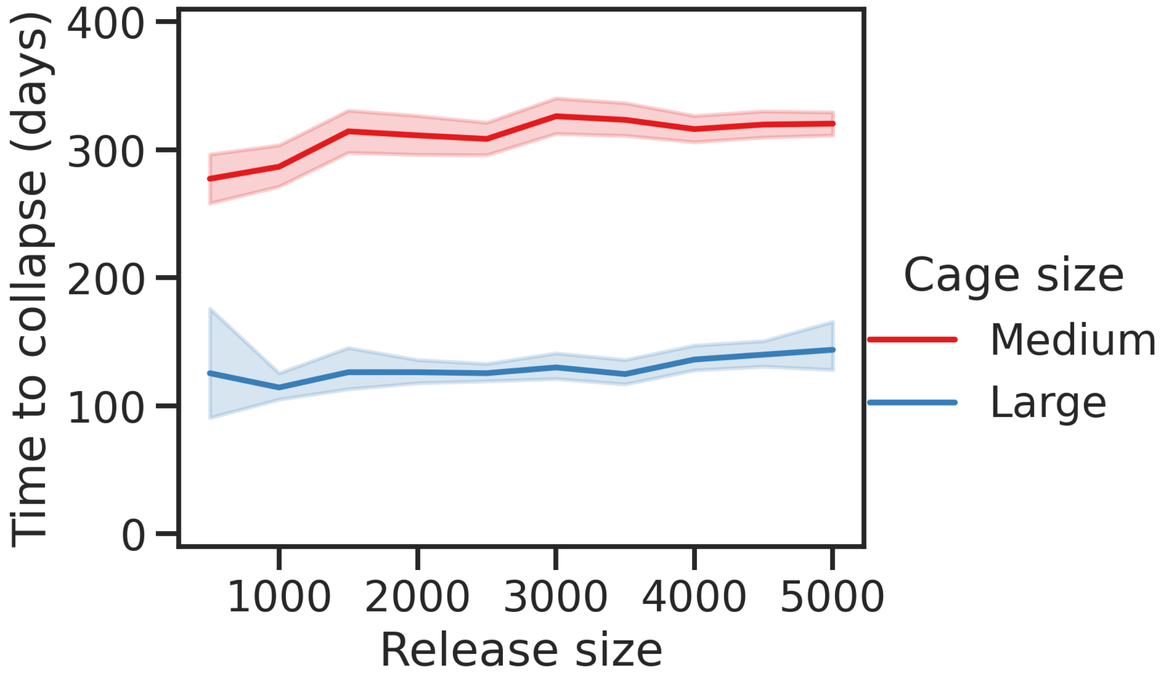

Supplementary Figure 5. Time required for population collapse after the introduction of the gene drive as a function of release size. For each release size, we have run 50 simulations, recording the time point at which the population size reaches zero. Time is measured from the first release of drive individuals. Solid lines indicate the average time, and the shaded areas indicate the 95% confidence interval.

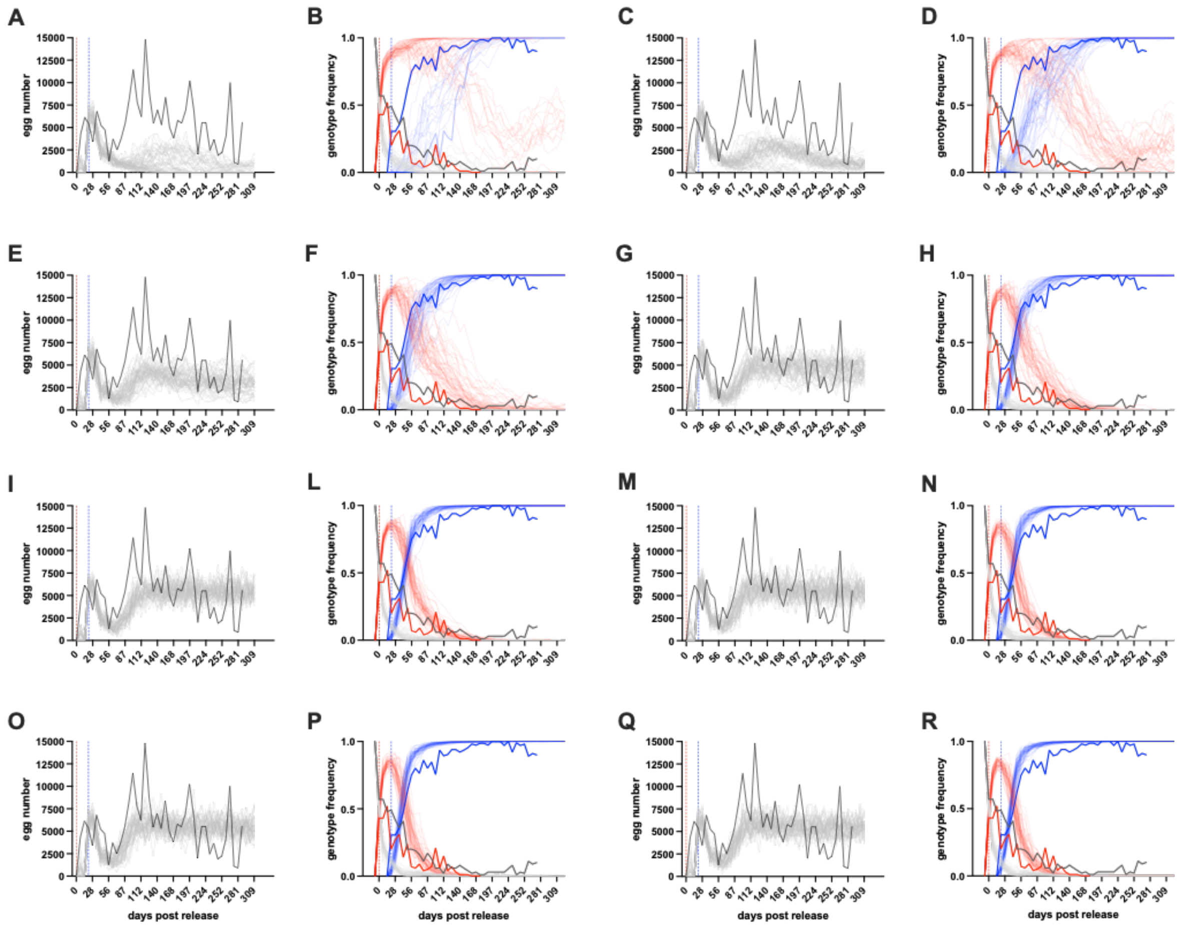

Supplementary Figure 6. Mathematical models of the population dynamics in the medium-sized cage using different mating probability values. For the 'gene drive + anti-drive' medium-sized cage, we have modelled the transgenic releases based on different mating probability values of Ag(Vasa:A4)$2^{+/+}$ males. In particular, egg number and genotype frequencies of wild type, gene drive and anti-drive are graphed as follows: mating probability value of 0.01 (graphs **A** and **B**), 0.05 (graphs **C** and **D**), 0.20 (graphs **E** and **F**), 0.30 (graphs **G** and **H**), 0.5 (graphs **I** and **L**), 0.7 (graphs **M** and **N**), 0.9 (graphs **O** and **P**), 1.0 (graphs **Q** and **R**).

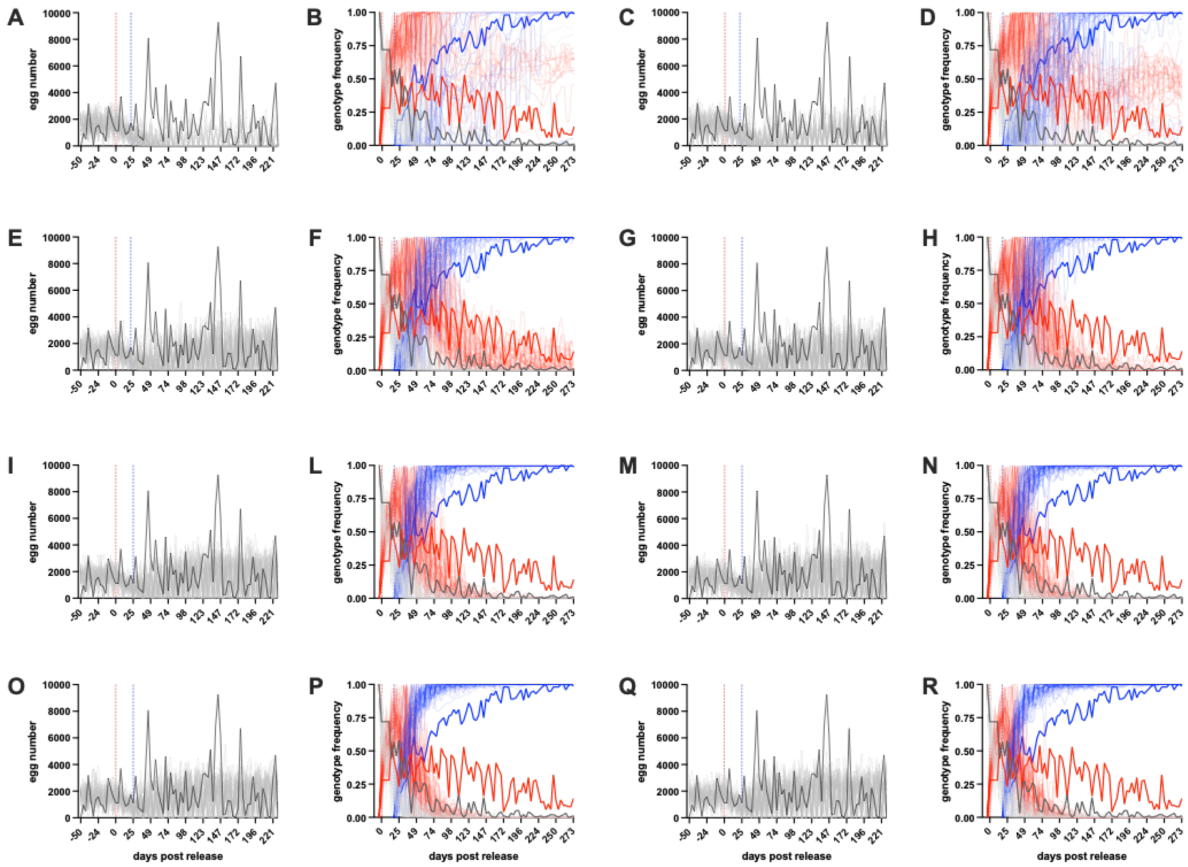

Supplementary Figure 7. Mathematical models of the population dynamics in the large-sized cage using different mating probability values. For the ‘gene drive + anti-drive’ large-sized cage, we have modelled the transgenic releases based on different mating probability values of Ag(Vasa:A4)2$^{+/+}$ males. In particular, egg number and genotype frequencies of wild type, gene drive and anti-drive are graphed as follows: mating probability value of 0.01 (graphs **A** and **B**), 0.05 (graphs **C** and **D**), 0.2 (graphs **E** and **F**), 0.3 (graphs **G** and **H**), 0.5 (graphs **I** and **L**), 0.7 (graphs **M** and **N**), 0.9 (graphs **O** and **P**), 1.0.

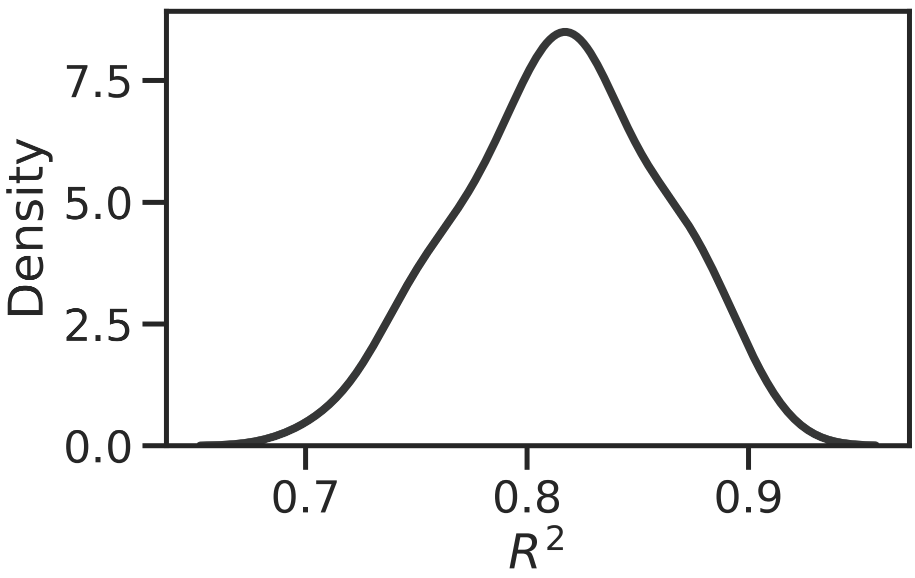

Supplementary Figure 8. Kernel density estimation distribution plot. Distribution (kernel density estimation) of R^2 values for goodness of fit for the chosen fitness parameters for the large cage populations, using the wild-type, gene drive and anti-drive genotype frequency. The distribution is determined from 50 iterations of the simulation.

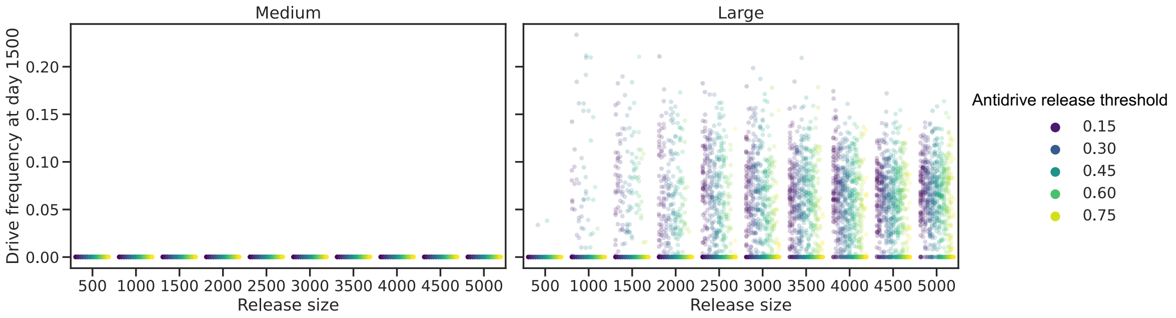

Supplementary Figure 9. Effect of population size and anti-drive release threshold over long-term gene drive dynamics. Gene drive frequency in the population after 1500 days of simulations, as a function of restocking release size (i.e. population size) and the initial gene drive frequency in the population at the time of anti-drive release (anti-drive release threshold) for medium sized (left hand panels) and large sized (right hand panels) cages. Each dot identifies a simulation for each release size and frequency threshold pair. Values 0 indicate the simulations where the gene drive was completely removed by the action of the anti-drive within 1500 days.

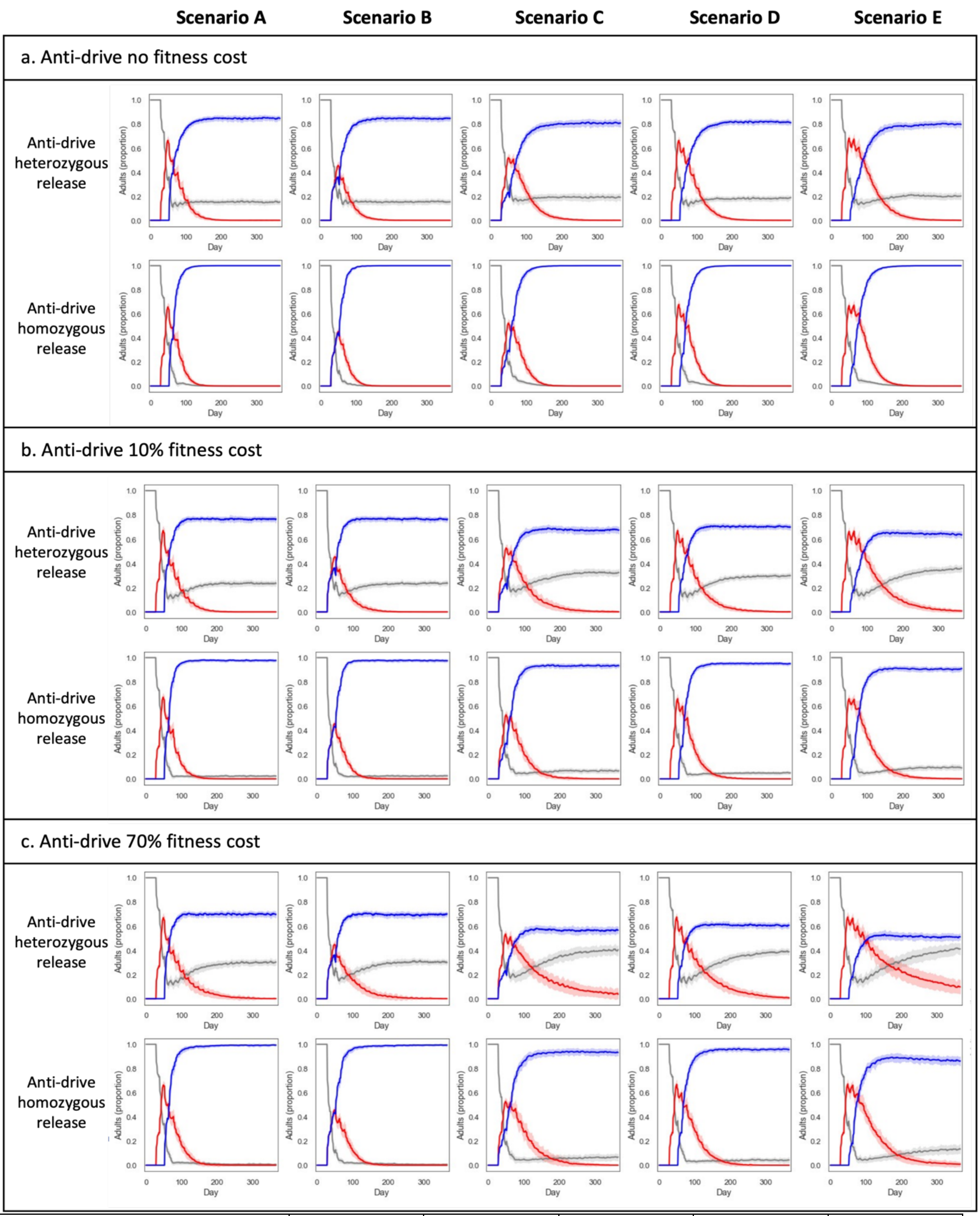

	A	B	C	D	E
Drive release frequency	50%, 6 releases	50%, 6 releases	50%, 6 releases	50%, 6 releases	50%, 6 releases
Anti-drive release frequency	50%	50%	30%	50%	30%
Simultaneous drive/anti-drive release	no	yes	yes	no	no
Continuous anti-drive release	yes	yes	yes	yes	yes
Release ‘on top’ or ‘within’ wildtype	within	within	within	on top	on top

Supplementary Figure 10. Preliminary modelling to estimate dynamics of drive and anti-drive individuals in large cage populations based on different release scenarios and fitness

costs. Drive (red), Anti-drive (blue) and wild-type (grey) adult mosquito proportions in simulated populations after five release scenarios (A-E). Each plot shows the average of fifty simulations and shaded area indicate standard deviation. In all scenarios, drive male mosquitoes are released at 50% of adult population frequency for 6 releases (twice a week for three weeks). Anti-drive mosquitoes are continuously released at 50% or 30% frequency (as indicated), simultaneously with the drive (simulation B and C) or after the six releases (simulations A, D, E). Release of Drive and Anti-drive were simulated 'on-top' of the restocking individuals (simulation D and E) or integrated 'within' the restocking individuals (simulations A, B and C). For each scenario, release of male heterozygous (top panels) or homozygous (bottom panels) anti-drive individuals were simulated. To explore genotype dynamic as a function of anti-drive fitness cost, each release scenario was simulated assuming a) no fitness cost associated to the anti-drive individual, b) 10% fitness cost associated to the anti-drive individuals or (c) 70% fitness cost associated to the anti-drive individuals. Fitness cost was assumed as a reduction of eggs.
