# Supplementary material for: Anti-CRISPR Anopheles mosquitoes inhibit gene drive spread under challenging behavioural conditions in large cages
Source: Nat Commun. 2024 Feb 1;15:952. doi: 10.1038/s41467-024-44907-x (PMC10830555; doi:10.1038/s41467-024-44907-x)
Supplement: Supplementary file 2 — Reporting Summary [file 41467_2024_44907_MOESM2_ESM.pdf]

nature portfolio

Corresponding author(s): Alekos Simoni a.simoni@imperial.ac.uk
Ruth Müller r.muller@itg.be

Last updated by author(s): Dec 15, 2023

Reporting Summary

Nature Portfolio wishes to improve the reproducibility of the work that we publish. This form provides structure for consistency and transparency in reporting. For further information on Nature Portfolio policies, see our Editorial Policies and the Editorial Policy Checklist.

Statistics

For all statistical analyses, confirm that the following items are present in the figure legend, table legend, main text, or Methods section.

n/a	Confirmed	
☐	☒	The exact sample size (n) for each experimental group/condition, given as a discrete number and unit of measurement
☐	☒	A statement on whether measurements were taken from distinct samples or whether the same sample was measured repeatedly
☐	☒	The statistical test(s) used AND whether they are one- or two-sided<br>*Only common tests should be described solely by name; describe more complex techniques in the Methods section.*
☒	☐	A description of all covariates tested
☐	☒	A description of any assumptions or corrections, such as tests of normality and adjustment for multiple comparisons
☐	☒	A full description of the statistical parameters including central tendency (e.g. means) or other basic estimates (e.g. regression coefficient) AND variation (e.g. standard deviation) or associated estimates of uncertainty (e.g. confidence intervals)
☐	☒	For null hypothesis testing, the test statistic (e.g. F, t, r) with confidence intervals, effect sizes, degrees of freedom and P value noted<br>*Give P values as exact values whenever suitable.*
☒	☐	For Bayesian analysis, information on the choice of priors and Markov chain Monte Carlo settings
☒	☐	For hierarchical and complex designs, identification of the appropriate level for tests and full reporting of outcomes
☒	☐	Estimates of effect sizes (e.g. Cohen's d, Pearson's r), indicating how they were calculated

Our web collection on statistics for biologists contains articles on many of the points above.

Software and code

Policy information about availability of computer code

Data collection	EggCounter v1.0 software was used for count eggs in the cage trials
Data analysis	GraphPad Prism 10.0 was used for statistical analysis and plotting graphs. CRISPResso2 was used to analyse results from amplicon sequencing. Numpu, scipy and pandas libraries were used for the mathematical model.

For manuscripts utilizing custom algorithms or software that are central to the research but not yet described in published literature, software must be made available to editors and reviewers. We strongly encourage code deposition in a community repository (e.g. GitHub). See the Nature Portfolio guidelines for submitting code & software for further information.

Data

Policy information about availability of data

All manuscripts must include a data availability statement. This statement should provide the following information, where applicable:

- Accession codes, unique identifiers, or web links for publicly available datasets
- A description of any restrictions on data availability
- For clinical datasets or third party data, please ensure that the statement adheres to our policy

Raw-sequencing data generated in this study have been deposited in the EBI-ENA database under accession code PRJEB61434 (https://www.ncbi.nlm.nih.gov/bioproject/?term=PRJEB61434) for the targeted nanopore sequencing, the whole genome nanopore sequencing and for the amplicon sequencing, while the hybrid AgamP4-Ag(Vasa:A4)2 reference genome is provided as a fasta file in the source data. The C119 plasmid sequence has been deposited in the GenBank database under accession code PRJEB61434 (https://www.ncbi.nlm.nih.gov/bioproject/?term=PRJEB61434). Source data are provided with this paper

Research involving human participants, their data, or biological material

Policy information about studies with human participants or human data. See also policy information about sex, gender (identity/presentation), and sexual orientation and race, ethnicity and racism.

Reporting on sex and gender	n/a
Reporting on race, ethnicity, or other socially relevant groupings	n/a
Population characteristics	n/a
Recruitment	n/a
Ethics oversight	n/a

Note that full information on the approval of the study protocol must also be provided in the manuscript.

Field-specific reporting

Please select the one below that is the best fit for your research. If you are not sure, read the appropriate sections before making your selection.

[x] Life sciences [ ] Behavioural & social sciences [ ] Ecological, evolutionary & environmental sciences

For a reference copy of the document with all sections, see nature.com/documents/nr-reporting-summary-flat.pdf

Life sciences study design

All studies must disclose on these points even when the disclosure is negative.

Sample size	Consistent with previous literature reporting similar phenotype assays (https://doi.org/10.1038/s41467-021-24790-6) the full progeny obtained from each oviposition/sample was counted/screened in each reported phenotype assay. Starting frequency, number of replicates and sampling used in th cage trial was consistent with previous literature reporting similar experiments (https://doi.org/10.1038/s41467-021-24790-6).
Data exclusions	No data exclusions.
Replication	Biological replicates were performed as described in the main text. Consistent with previous literature (https://doi.org/10.1038/s41467-021-24790-6) a minimum of seven biologically independent samples (ovipositing females) were examined over 2 independent experiments for each cross/experiment. All attempts of replication were successful.
Randomization	For the cage trial randomization was applied when selecting eggs to seed consecutive generations according to previous literature (https://doi.org/10.1038/s41467-021-24790-6)
Blinding	Blinding was not relevant to this study because none of the data recorded were subjective (i.e., number of larvae, eggs or mosquitoes positive for specific/unequivocal fluorescent markers).

Reporting for specific materials, systems and methods

We require information from authors about some types of materials, experimental systems and methods used in many studies. Here, indicate whether each material, system or method listed is relevant to your study. If you are not sure if a list item applies to your research, read the appropriate section before selecting a response.

Materials & experimental systems

n/a	Involved in the study
☒	☐ Antibodies
☒	☐ Eukaryotic cell lines
☒	☐ Palaeontology and archaeology
☐	☒ Animals and other organisms
☒	☐ Clinical data
☒	☐ Dual use research of concern
☒	☐ Plants

Methods

n/a	Involved in the study
☒	☐ ChIP-seq
☒	☐ Flow cytometry
☒	☐ MRI-based neuroimaging

Animals and other research organisms

Policy information about studies involving animals; ARRIVE guidelines recommended for reporting animal research, and Sex and Gender in Research

Laboratory animals	The Anopheles gambiae G3 wild-type stock used in this study, originally procured from BEI Resources (MRA-112), has been in culture for over 30 years. The strain Ag(QFS)1 used in the study has been previously described (Kyrou et al 2018). The strain Ag(Vasa:A4) used in this study has been previously described in Taxiarchi et al.,2019. Housing and experimental conditions followed standard procedures as described in the manuscript. Phenotype assays were performed with mosquitoes 3-5 days old. Sex of tested mosquitoes is indicated for each assay. Both sexes were included.
Wild animals	The study did not involve wild animals.
Reporting on sex	Both male and female sexes were included in this study, as indicated in each assay.
Field-collected samples	The study did not involve samples collected from the field.
Ethics oversight	All animal work was conducted according to the Italian regulations at Polo GGB and according to UK Home Office Regulations at Imperial College London.

Note that full information on the approval of the study protocol must also be provided in the manuscript.

Plants

Seed stocks	n/a
Novel plant genotypes	n/a
Authentication	n/a
